# Inflammatory bowel disease and bladder cancer risk: based on a Mendelian randomization study

**DOI:** 10.1186/s12894-023-01346-y

**Published:** 2023-11-27

**Authors:** Li Wang, Jing-ya Deng, Kun-peng Li, Shan-Yin, Ping-yu Zhu

**Affiliations:** 1 Department of Urology, Affiliated Hospital of North Sichuan Medical College, Nanchong, China; 2 Department of Neurology, Affiliated Hospital of North Sichuan Medical College, Nanchong, China; 3 Department of Urology, The Second Hospital of Lanzhou University, Lanzhou, China

**Keywords:** Mendelian randomization, Bladder cancer, Inflammatory bowel Disease, Crohn’s Disease, Ulcerative Colitis

## Abstract

**Background:**

Prior epidemiological observational studies have duly documented a correlative link between inflammatory bowel disease (IBD) and bladder cancer (BC); however, the establishment of a definitive causal relationship has remained elusive. The principal objective of this meticulous investigation was to rigorously evaluate the causal nexus between IBD and BC, employing the robust methodology of Mendelian randomization (MR) analysis.

**Methods:**

We meticulously performed both univariate and multivariate Mendelian randomization (MVMR) analyses employing publicly accessible genome-wide association study (GWAS) data. The central approach employed for our investigations was inverse variance weighting (IVW) method, while diligently scrutinizing potential sources of heterogeneity and horizontal pleiotropy via the rigorous utilization of Cochran’s Q test, the MR-PRESSO method, and MR-Egger.

**Results:**

In the univariate MR analysis, no causal link was observed between genetic prediction of IBD and BC. Furthermore, both Crohn’s disease (CD) and ulcerative colitis (UC) showed no causal association with BC. The consistent association between CD and UC in the MVMR analysis supports this finding.

**Conclusion:**

This study found no genetic basis for the causative association of IBD and BC. It is crucial to emphasize that further comprehensive investigations are warranted to delve into the intricate underlying mechanisms that may contribute to these associations.

**Supplementary Information:**

The online version contains supplementary material available at 10.1186/s12894-023-01346-y.

## Introduction

Bladder cancer (BC) is a prevalent global malignancy, ranking as the tenth most common cancer worldwide. Its high recurrence rate contributes to its status as the most expensive cancer to treat over a lifetime [1]. Globally, it ranks thirteenth in both incidence and mortality, with a striking 123.34% increase in cases from 1990 to 2019 [2, 3]. Risk factors include exposure to toxic chemicals and smoking. Given its significant burden, identifying modifiable risk factors to reduce its incidence is crucial.

Inflammatory bowel disease (IBD), which encompasses ulcerative colitis (UC) and Crohn’s disease (CD), represents a chronic and recurrent inflammatory disorder attributed to aberrations in the immune system within the intestinal mucosa. This intricate interplay leads to the manifestation of extraintestinal symptoms and immune dysfunction [4]. Of grave concern is the escalating prevalence and incidence of IBD, casting a global shadow over public health, affecting over 1.5 million individuals in North America and exceeding 2 million in Europe [5]. Notably, inflammation assumes a pivotal role in the pathogenesis of tumorigenesis, bestowing upon those afflicted with IBD an augmented susceptibility to the development of both intestinal and extraintestinal neoplasms [6, 7]. Given the anatomical adjacency of the bowel and bladder, insightful epidemiological studies have endeavored to elucidate the potential association between IBD and the risk of bladder cancer. Regrettably, the findings have yielded a landscape of incongruous outcomes [8, 9].

Traditional observational studies are susceptible to biases from confounding factors and reverse causality. Mendelian randomization (MR) provides a quasi-randomized controlled trial (RCT) design, using single nucleotide polymorphisms (SNPs) as exposure indicators to establish causal associations and genetic variation as instrumental variables (IVs) to reduce confounding [10]. We employed univariate and multivariate Mendelian randomization (MVMR) using summary statistics from genome-wide association studies to investigate causal links between IBD and BC.

## Methods

**Fig. 1:**
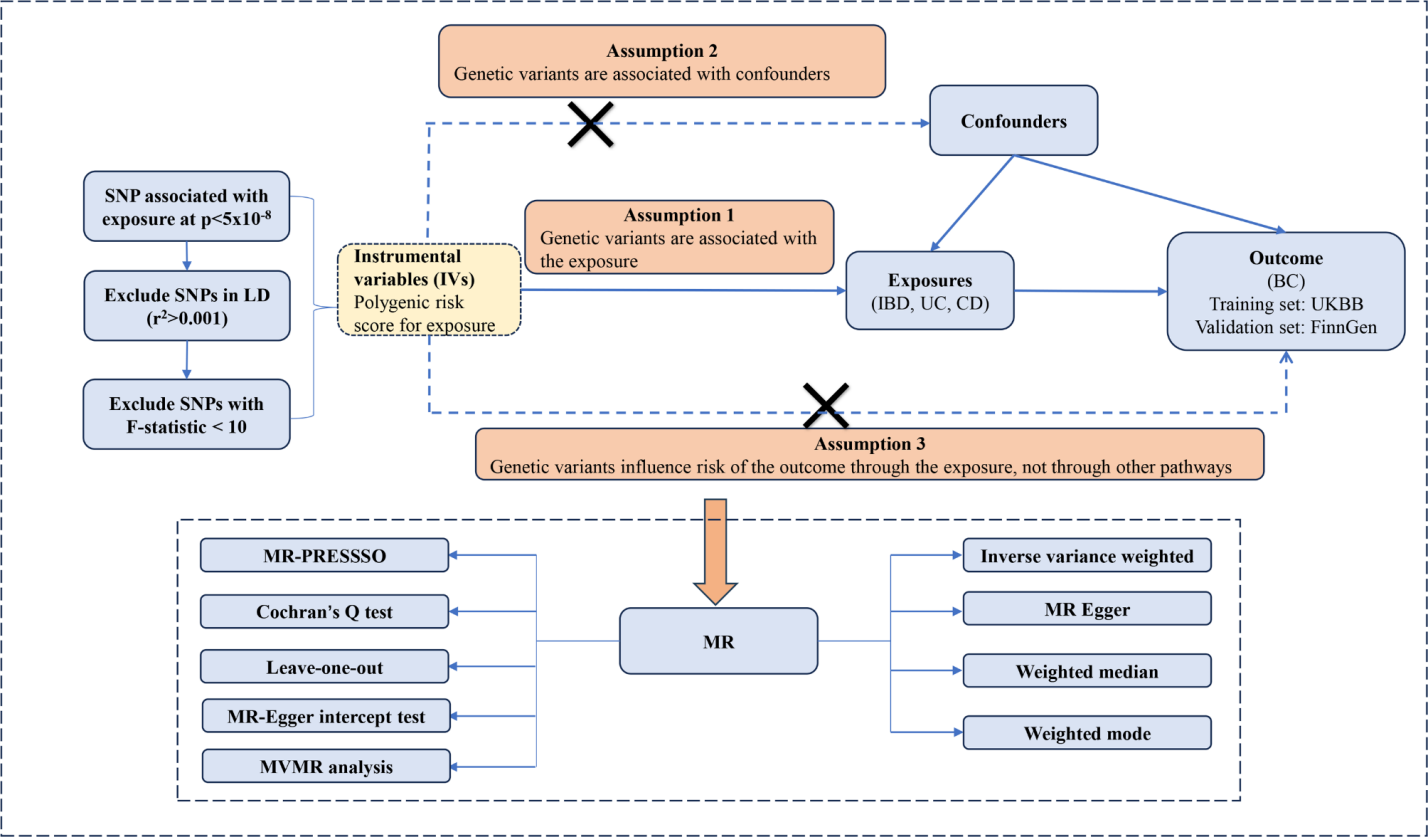
Study flow chart

The study strictly followed the guidelines outlined in the Strengthening the Reporting of Observational Studies in Epidemiology Mendelian randomization (STROBE-MR) framework [11]. MR relies on three essential assumptions: IVs demonstrate strong correlation with exposure factors, remain unaffected by confounding variables, and impact outcomes solely through the exposure under investigation [12]. Our study employed a two-sample MR and MVMR approach, adhering to these assumptions and the methods delineated in Fig. 1.

### Data sources

Specific details regarding the genetic datasets utilized in this study can be found in Table 1. The GWAS dataset for European ancestry individuals with IBD, encompassing both CD and UC, was obtained from the International Inflammatory Bowel Disease Genetics Consortium (IIBDGC). The IBD dataset comprised 31,665 cases and 33,977 controls, with UC accounting for 13,768 cases and CD accounting for 17,897 cases, all of which were matched with 33,977 controls [13]. The diagnostic criteria for this disease type depend on established endoscopic, imaging, and histological pathology standards [14].

**Table 1:** Details of studies and datasets used for analyses

Exposure or outcome	Study or consortium	Participants(case/control)	Ethnicity	PubMed ID or web source
Inflammatory bowel disease	IIBDGC	31,665/33,977	European	26,192,919
Crohn’s disease	IIBDGC	17,897/33,977	European	26,192,919
Ulcerative colitis	IIBDGC	13,768/33,977	European	26,192,919
Bladder cancer	United Kingdom Biobank	2,576/ 417,955	European	https://pan-ukb-us-east-1.s3.amazonaws.com/sumstats_flat_files/icd10-C67-both_sexes.tsv.bgz
Bladder cancer	FinnGen	2,053/287,137	European	https://storage.googleapis.com/finngen-public-data-r9/summary_stats/finngen_R9_C3_BLADDER_EXALLC.gz

IIBDGC: International Inflammatory Bowel Disease Genetics Consortium

The GWAS data from the United Kingdom Biobank (UKBB) served as the training set for bladder cancer (2,576 cases and 417,955 controls) (https://www.ukbiobank.ac.uk/). Bladder cancer cases were identified using the International Classification of Diseases (ICD-9 and ICD-10) codes. Additionally, to reduce bias caused by sample overlap, the validation set consisted of GWAS data from the FinnGen database (R9 version) with 2,053 cases and 287,137 controls (https://r9.finngen.fi/). Logistic regression calculations were performed to adjust for genetic effect sizes, as well as age, sex, and genetic principal components, within the Finnish population.

### Selection of instrumental variables

To ensure the stability of the causal relationship between IBD, including UC and CD, and BC, IVs were selected based on the following principles: [1] Significant SNPs with genetic effects on IBD (UC or CD) were chosen as IVs (p < 10^− 8^) [2]. Cluster analysis was conducted to address linkage disequilibrium (LD) among the selected IVs (r^2^ < 0.001, kb = 10,000) [3]. To mitigate bias from weak IVs, the strength of the IVs was quantified using the F value (β^2^/SE), with those having F < 10 being excluded. Here, β represents the effect size of exposure and SE represents the standard error of the effect size [4]. The identified SNPs were cross-referenced with the Phenoscanner database to account for potential confounders such as smoking. However, due to the presence of shared genes between CD and UC, MVMR was performed to mitigate any mutual bias caused by this overlap.

### MR analysis

To obtain preliminary causal estimates, a comprehensive two-sample MR analysis was conducted. The primary analysis employed the robust inverse-variance weighted (IVW) method [15]. In addition, we employed the weighted median, MR-Egger regression, and weighted mode methods as validation approaches. The weighted median method, known for its reliability, yielded dependable results by leveraging a powerful instrumental variable with a weight of 50% [16]. To address potential directional pleiotropy, MR-Egger regression and weighted mode methods were employed [17, 18]. To assess the independent effects of traits associated with UC and CD, we conducted MVMR analyses. We employed IVW and LASSO regression methods to evaluate the MVMR results. in addition, potential inverse associations were avoided by the Setiger test [19]. The statistical power calculations were performed using the mRnd website (https://shiny.cnsgenomics.com/mRnd/) [20].

Sensitivity analysis assumes a vital role in the assessment of heterogeneity and potential biases within MR studies. Firstly, heterogeneity was evaluated through the application of Cochran’s Q test, which involved calculating the weighted sum of squared differences between specific variability estimates and the overall IVW estimate [21]. To address potential outliers, the MR Pleiotropy RESidual Sum and Outlier (MR-PRESSO) method was employed during data analysis [22]. Furthermore, MR-Egger regression was utilized, and intercepts were assessed to identify potential horizontal pleiotropy. The leave-one-out method was implemented to mitigate the impact of individual SNPs causing horizontal pleiotropy.

### Statistical analysis

The statistical analyses were conducted using the “TwoSampleMR” and “MRPRESSO” packages in R version 4.2.2. For visual representation, the “forest plot” package was employed. The association IBD and BC in the MR analysis was quantified using odds ratios (OR) with corresponding 95% confidence intervals (CI). To account for multiple testing between IBD (UC and CD) and BC, a significance level of p < 0.025 (0.05/2 databases) was applied after Bonferroni correction. Results with 0.025 ≤ p < 0.05 were considered suggestive of significance.

## Results

### Selection of genetic variants

First analyzed in the training group, the bladder cancer dataset from UK Biobank, after harmonization and removal of palindromic SNPs with intermediate allele frequencies, we further eliminated confounding SNPs (rs181826, rs3184504, rs6062496, rs13407913) using the Phenoscanner database. In addition, outliers are detected and removed by MR–PRESSO. Subsequently, we identified 126 SNPs as potential instrumental variables (IVs) for IBD, with 113 SNPs specific to CD and 84 SNPs specific to UC. Additional file 1 provides detailed information on these SNPs. When using the FinnGen dataset validation, we identified 124 SNPs as potential instrumental variables (IVs) for IBD, with 114 SNPs specific to CD and 76 SNPs specific to UC (Additional file 2). Importantly, all the included SNPs had F-values exceeding 10, indicating a minimal likelihood of weak IVs bias. The results of power analyses were shown in Table 2.

**Table 2:** Multivariable MR analysis between IBD and BC

Exposure	Outcome	No. of SNPs	IVW				LASSO regression		
			Beta	SE	Pvalue		Beta	SE	Pvalue
Training set	UKBB								
CD	BC	137	0.04	0.03	0.27		0.02	0.03	0.39
UC			-0.05	0.04	0.24		-0.02	0.04	0.55
Validation set	FinnGen								
CD	BC	132	-0.05	0.04	0.22		-0.03	0.04	0.32
UC			0.09	0.05	0.04		0.09	0.05	0.03

UC: ulcerative colitis; CD: Crohn’s disease; IVW: inverse variance weighted; UKBB: United Kingdom Biobank

### A causal association between IBD and BC

**Fig. 2:**
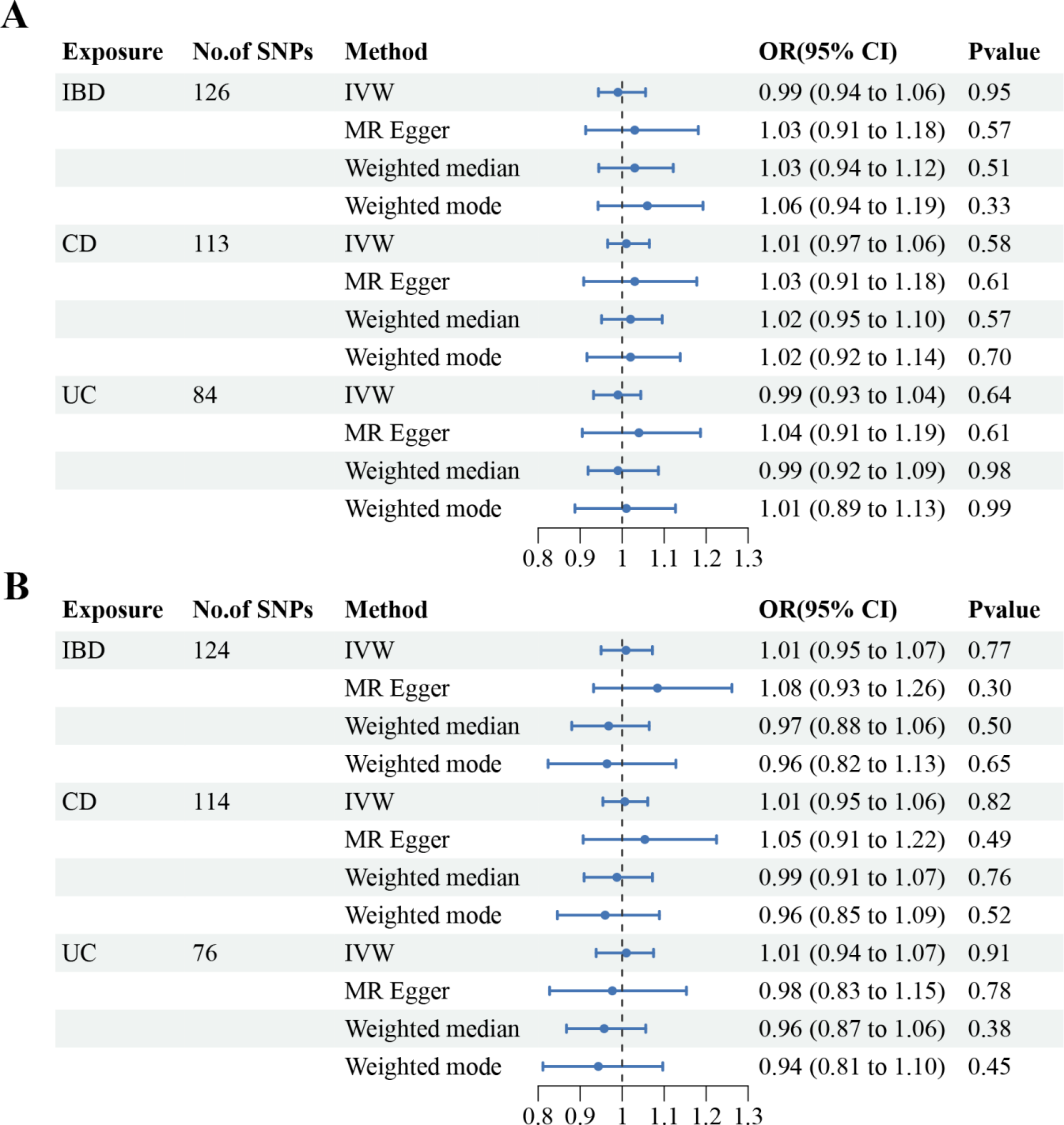
Results of the methods of MR analysis conducted to examine the relationship between IBD and BC. **(A)** UKBB test Group **(B)** FinnGen validation group

In the test group, MR results from UKBB showed no causal link IBD (CD and UC) and BC (For IBD, IVW OR 0.99, 95% CI 0.94 to 1.06, p = 0.95; For CD, IVW OR 1.01, 95%CI 0.97 to 1.06, p = 0.58; For UC, IVW OR 0.99, 95% CI 0.93 to 1.04, p = 0.64) (Fig. 2A). In addition, MR results from the FinnGen validation group showed no causal association between IBD (CD and UC) and BC (For IBD, IVW OR 1.01, 95% CI 0.95 to 1.07, p = 0.77; For CD, IVW OR 1.01, 95%CI 0.95 to 1.06, p = 0.82; For UC, IVW OR 1.01, 95% CI 0.94 to 1.07, p = 0.91). and the MR-Egger, the Weighted Median, and the Weighted mode showed consistent results (Fig. 2B). Considering the interrelated nature of CD and UC, a multivariate Mendelian randomization (MVMR) analysis was conducted to assess the individual impact of both diseases on BC. After adjusting for each other using IVW, the results indicated no causal relationship between IBD and BC. The reliability of these findings was further supported by LASSO regression analysis (Table 3).

**Table 3:** Sensitivity analysis of the causal association between IBD and BC

Exposure	Outcome		Heterogeneity			Horizontal pleiotropy			
			Cochran Q statistic			MR-Egger		MR–PRESSO (Outlier-corrected)	
		IVW Q	IVW *I*^*2*^	IVW *P*^#^		Egger intercept	*P* ^#^ *value*	Outlier	P^*^ for global test
Training set	UKBB								
IBD	BC	147.9	15.48	0.08		-0.004	0.5	“rs7608910”,“rs1569328”	0.07
CD		131.2	14.6	0.1		-0.002	0.74	“rs1569328”,“rs7608910”	0.11
UC		90.3	8.11	0.27		-0.006	0.43	0	0.26
Validation set	Finngen								
IBD	BC	131.4	6.45	0.28		-0.008	0.3	0	0.27
CD		119.8	5.74	0.31		-0.006	0.51	0	0.3
UC		71.1	0	0.6		0.003	0.72	“rs12132349”,“rs36070529”,“rs4795397”,“rs9271255”	0.6

* MR analysis using IVW method after removing outliers identified by MR-PRESSO method

# Heterogeneity test after removing outliers

IBD, inflammatory bowel disease; CD, Crohn’s disease; UC, ulcerative colitis; BC, bladder cancer; IVW, inverse variance weighted; OR, odds ratio; CI, confidence interval; SE, standard error

### Pleiotropic and heterogeneity analysis

In UKB consortium data, The MR-Egger method suggested that there was no evidence of horizontal pleiotropy in IBD (egger intercept = -0.004, p = 0.5), CD (egger intercept = -0.002, p = 0.74), UC (egger intercept = -0.006, p = 0.43). In the FinnGen validation dataset, horizontal pleiotropy was also not detected (Table 4). Based on the results of MR-PRESSO, the outlier instrumental variables have been removed (global P > 0.05). The Cochran’s Q test revealed no significant heterogeneity among the selected genetic instruments (P > 0.05), allowing us to utilize a fixed effects model to estimate the effect size in the Mendelian randomization (MR) analysis. The robustness and stability of the results were further confirmed through the implementation of leave-one-out analysis and the visualization of forest plots (Additional file 3). These findings indicate the reliability and consistency of the current MR analysis.

**Table 4:** Power calculation for all the MR analysis in current study

Exposure	Outcome	No. of SNPs	Proportion of variance explained by the SNPs on exposure	Power (%)
Training set	UKBB			
IBD	BC	126	0.59	17%
CD		113	0.76	16%
UC		84	0.53	11%
Validation set	FinnGen			
IBD	BC	124	0.69	20%
CD		114	0.75	15%
UC		76	0.45	10%

## Discussion

To the best of our knowledge, this study represents a pioneering investigation into the potential causal relationship between IBD and its subtypes with regards to BC using pooled data from GWAS. Through the rigorous application of MR analysis, our findings do not provide potential evidence supporting a causal association between genetically predicted IBD and its subtypes, and the risk of BC.

Two distinct diseases within the same individual may stem from shared environmental or genetic factors. The mechanisms underlying inflammation-related carcinogenesis primarily involve the induction of epigenetic alterations and DNA damage [23]. IBD is known to be associated with long-term complications, including the development of gastrointestinal and extraintestinal tumors. Previous epidemiological studies have established a strong link between IBD and an elevated risk of various cancers (e.g., hematological, lung and non-Hodgkin’s lymphoma) [24]. However, the definitive evidence supporting an association between IBD, and BC risk remains inconclusive.

Kappelman et al. [25] conducted a comprehensive study with a follow-up period spanning over 30 years and reported a slight increase in the risk of BC among patients with CD (standardized incidence rate [SIR]: 1.1; 95% CI: 0.8 to 1.6). Furthermore, it has been observed that individuals with IBD, including CD, are at a higher risk of developing malignancies related to smoking. In a study by Madanchi et al. [26], which examined the incidence of malignancies in IBD centers over a 7-year period, a higher incidence of bladder cancer was noted in IBD patients compared to those without IBD (21.7/100,000). However, Algaba et al. [27], in a cohort study, did not find a significant overall increase in the risk of cancer among individuals with IBD, although the risk of bladder cancer remained elevated (relative risk [RR]: 5.23; 95% CI: 1.95–13.87). Conversely, Pedersen et al. [8]observed no difference in the risk of bladder cancer between patients with IBD and the general population (SIR: 0.99; 95% CI: 0.63–1.54).

MR did not reveal a potential causal association between IBD and its subtypes and BC. The inconsistent results obtained in previous observational studies can be explained in several ways. Firstly, inflammation has a synergistic effect with bladder carcinogenesis, with IBD-related inflammation accumulating in the rectum and surrounding organs (prostate, bladder), and the oxidative stress process of chronic inflammation is one of the potential risk factors promoting bladder carcinogenesis [28]. In a recent study in the field of information biology [29], notable findings have emerged, unveiling a substantial upregulation of Aurora kinases B, Cyclin-dependent kinases-1, and Cyclin A2 expression levels within the bladder tissues of patients afflicted by bladder cancer (BC) and the intestinal tissues of those suffering from inflammatory bowel disease (IBD). it suggests that the heightened activity within the cell cycle pathway may potentially serve as a catalyst for the advancement of immune responses in the context of both these debilitating conditions, thereby intricately interweaving the trajectories of BC and IBD. Furthermore, pertinent immunological analyses have elucidated the plausible involvement of three distinct genes in the orchestration of immune homeostasis, governing the intricate dynamics of inflammatory cell infiltration [29, 30]. Secondly, alterations in the intestinal flora are common in patients with IBD, and changes in the microbiological environment have been shown to be associated with cancer development [31]. Additionally, smoking is a significant causative factor in BC. Interestingly, smoking is believed to increase the risk of developing CD while improving the course of UC [32, 33].

Moreover, it is worth noting that immunosuppression, a common treatment approach for IBD, has been associated with an elevated overall risk of cancer. A retrospective study revealed a substantial threefold increase in the risk of urothelial cancer among IBD patients who were exposed to thiopurines, a class of immunosuppressive medications [34]. Immunosuppression promotes the proliferation of both EBV and HPV-infected cells, and Sun et al. [35] revealed a causal link between HPV and bladder cancer through MR analysis. Of course, future attention needs to be paid to the effect of drug dose, type, and duration of use on tumors.

### Strength and limitation

Our MR analysis has the following advantages. Firstly, it represents the first large-scale utilization of GWAS data to infer the causal relationship between IBD and bladder cancer, thus reducing confounding factors and reverse bias. Secondly, the study population comprised individuals of European origin, minimizing population stratification interference. Lastly, sensitivity analyses and diverse model estimates were employed to ensure result reliability. However, certain limitations cannot be avoided. Firstly, the findings require validation in other populations. Secondly, the course of IBD is unpredictable, characterized by alternating relapses and remissions, and patient medication usage remains unknown. Finally, future investigations with larger sample sizes and precise stratified analyses are necessary to confirm the underlying physiopathological mechanisms.

## Conclusion

In summary, this study found no genetic basis for the causative association of IBD and BC, it is essential to emphasize the need for further investigation and confirmation of this potential association in future research.

### Electronic supplementary material

Below is the link to the electronic supplementary material.

Additional file 1. Associations of single nucleotide polymorphisms for Inflammatory Bowel Disease in UK biobank.

Additional file 2. Associations of single nucleotide polymorphisms for Inflammatory Bowel Disease in FinnGen.

Additional file 3. **Figure ****S1**. Scatter plot (A), funnel plot (B), leave-one-out sensitivity analysis (C), forest plot (D) of the causal effect of IBD on BC risk based on UKBB. **Figure ****S2**. Scatter plot (A), funnel plot (B), leave-one-out sensitivity analysis (C), forest plot (D) of the causal effect of CD on BC risk based on UKBB. **Figure ****S3**. Scatter plot (A), funnel plot (B), leave-one-out sensitivity analysis (C), forest plot (D) of the causal effect of UC on BC risk based on UKBB. **Figure S4**. Scatter plot (A), funnel plot (B), leave-one-out sensitivity analysis (C), forest plot (D) of the causal effect of IBD on BC risk based on FinnGen. **Figure S5**. Scatter plot (A), funnel plot (B), leave-one-out sensitivity analysis (C), forest plot (D) of the causal effect of CD on BC risk based on FinnGen. **Figure S6**. Scatter plot (A), funnel plot (B), leave-one-out sensitivity analysis (C), forest plot (D) of the causal effect of UC on BC risk based on FinnGen.

## Data Availability

The datasets analyzed in this study are from the following datasets: (https://gwas.mrcieu.ac.uk/), (r9.finngen.fi) and (https://www.ukbiobank.ac.uk/), See Table 1 for more details.

## Abbreviations

IBD: Inflammatory bowel disease
BC: Bladder cancer
MR: Mendelian randomization
MVMR: Multivariate Mendelian randomization
GWAS: Genome-wide association study
IVW: Inverse variance weighting
CD: Crohn’s disease
UC: Ulcerative colitis
RCT: Randomized controlled trial
SNPs: Single nucleotide polymorphisms
IVs: Instrumental variables
STROBE-MR: Strengthening the Reporting of Observational Studies in Epidemiology Mendelian randomization
IIBDGC: International Inflammatory Bowel Disease Genetics Consortium
UKBB: United Kingdom Biobank
ICD: International Classification of Diseases
LD: Linkage disequilibrium
MR-PRESSO: MR Pleiotropy RESidual Sum and Outlier
OR: Odds ratios
