# Supplementary material for: Inflammatory bowel disease and bladder cancer risk: based on a Mendelian randomization study
Source: BMC Urol. 2023 Nov 27;23:195. doi: 10.1186/s12894-023-01346-y (PMC10683281; doi:10.1186/s12894-023-01346-y)
Supplement: Supplementary file 3 — Additional file 3. Figure S1. Scatter plot (A), funnel plot (B), leave-one-out sensitivity analysis (C), forest plot (D) of the causal effect of IBD on BC risk based on UKBB. Figure S2. Scatter plot (A), funnel plot (B), leave-one-out sensitivity analysis (C), forest plot (D) of the causal effect of CD on BC risk based on UKBB. Figure S3. Scatter plot (A), funnel plot (B), leave-one-out sensitivity analysis (C), forest plot (D) of the causal effect of UC on BC risk based on UKBB. Figure S4. Scatter plot (A), funnel plot (B), leave-one-out sensitivity analysis (C), forest plot (D) of the causal effect of IBD on BC risk based on FinnGen. Figure S5. Scatter plot (A), funnel plot (B), leave-one-out sensitivity analysis (C), forest plot (D) of the causal effect of CD on BC risk based on FinnGen. Figure S6. Scatter plot (A), funnel plot (B), leave-one-out sensitivity analysis (C), forest plot (D) of the causal effect of UC on BC risk based on FinnGen. [file 12894_2023_1346_MOESM3_ESM.pdf]

Supplementary Figure S1.

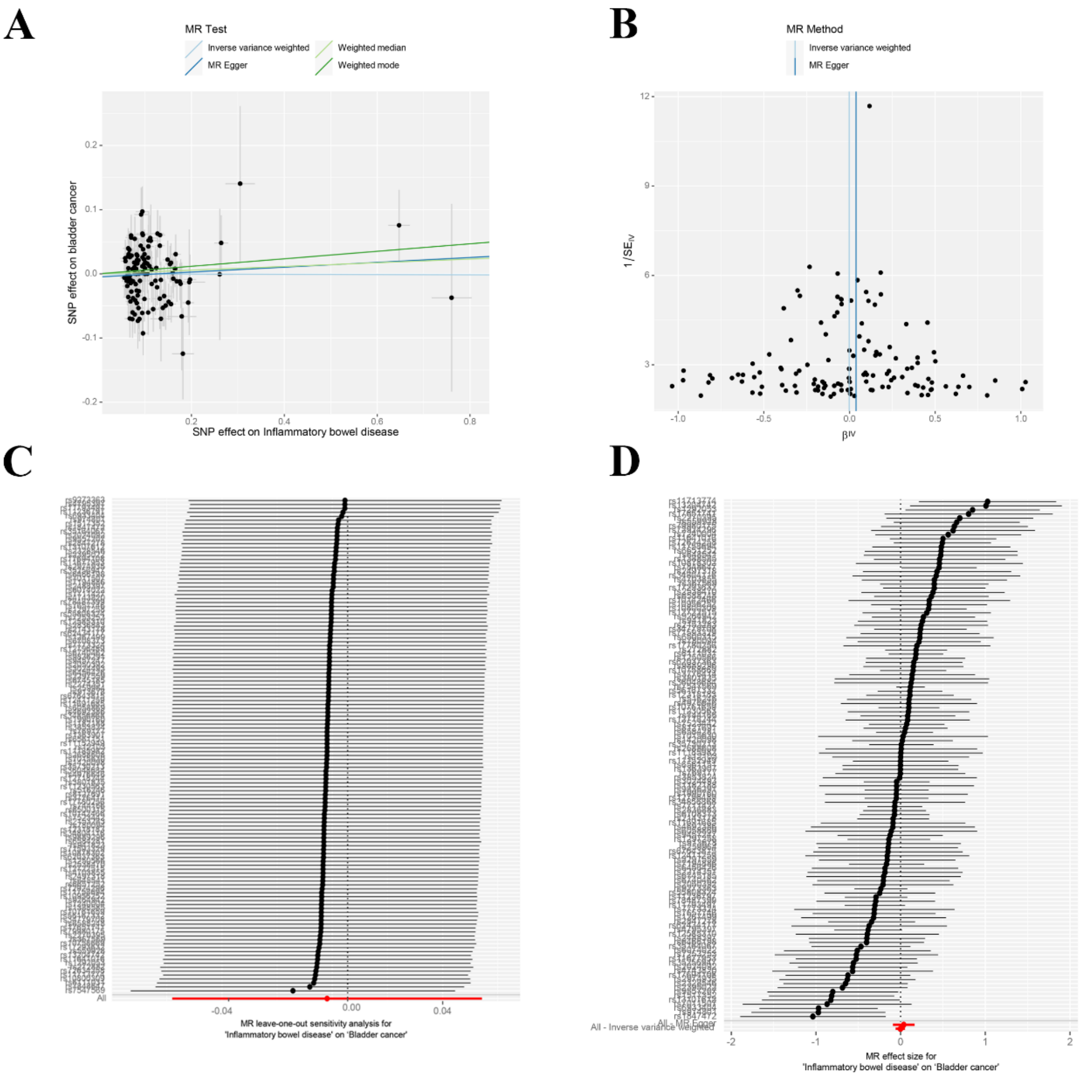

Figure S1. Scatter plot (A), funnel plot (B), leave-one-out sensitivity analysis (C), forest plot (D) of the causal effect of IBD on BC risk based on UKBB.

Supplementary Figure S2.

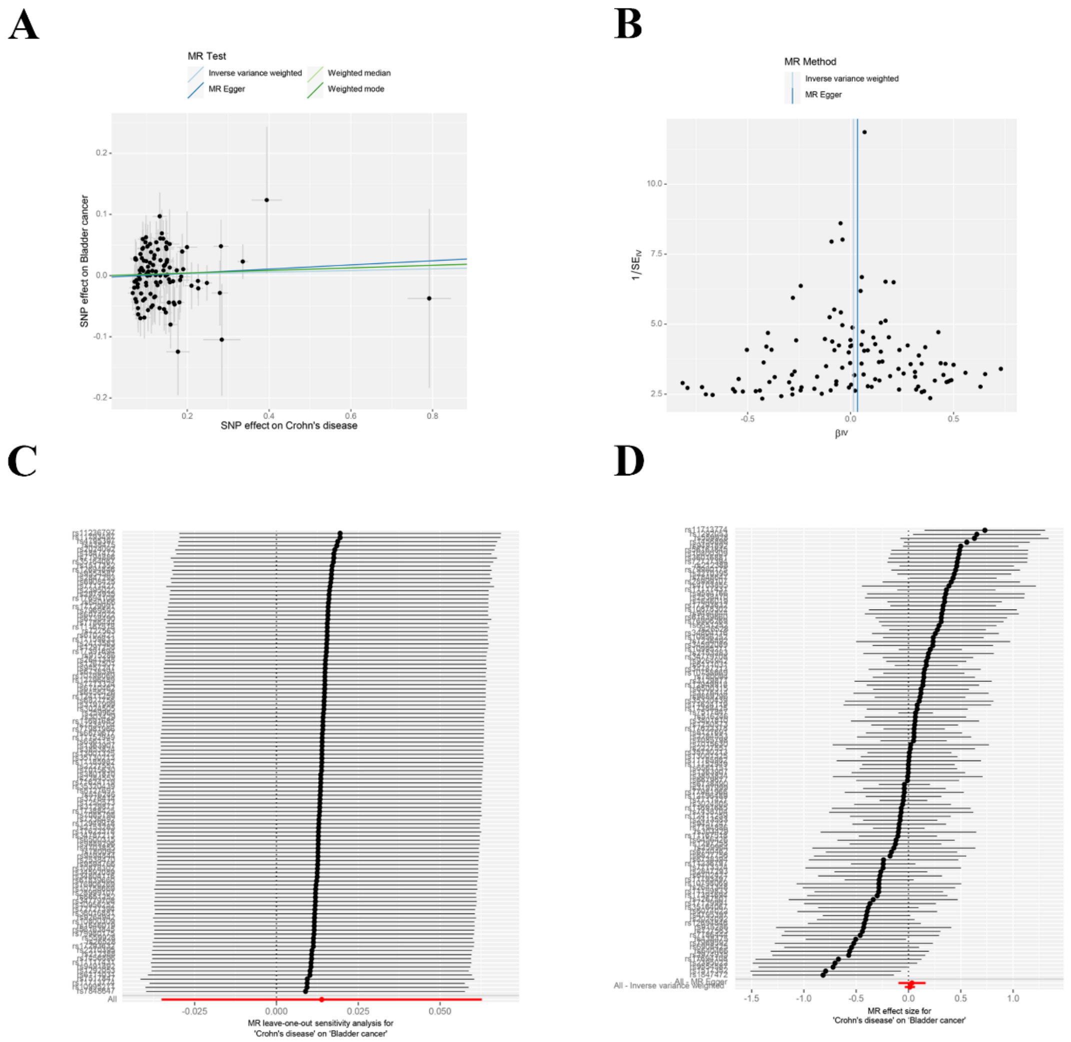

Figure S2. Scatter plot (A), funnel plot (B), leave-one-out sensitivity analysis (C), forest plot (D) of the causal effect of CD on BC risk based on UKBB.

Supplementary Figure S3.

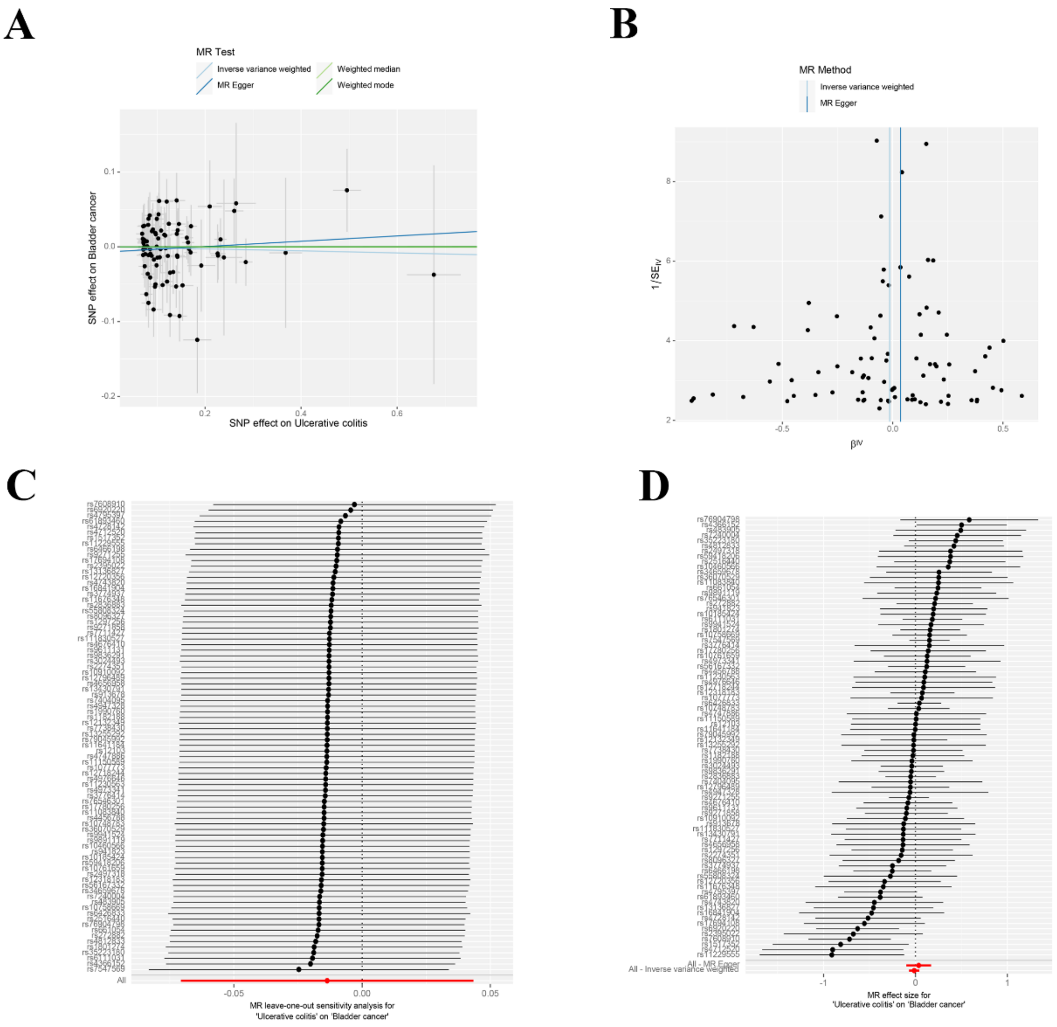

Figure S3. Scatter plot (A), funnel plot (B), leave-one-out sensitivity analysis (C), forest plot (D) of the causal effect of UC on BC risk based on UKBB.

Supplementary Figure S4.

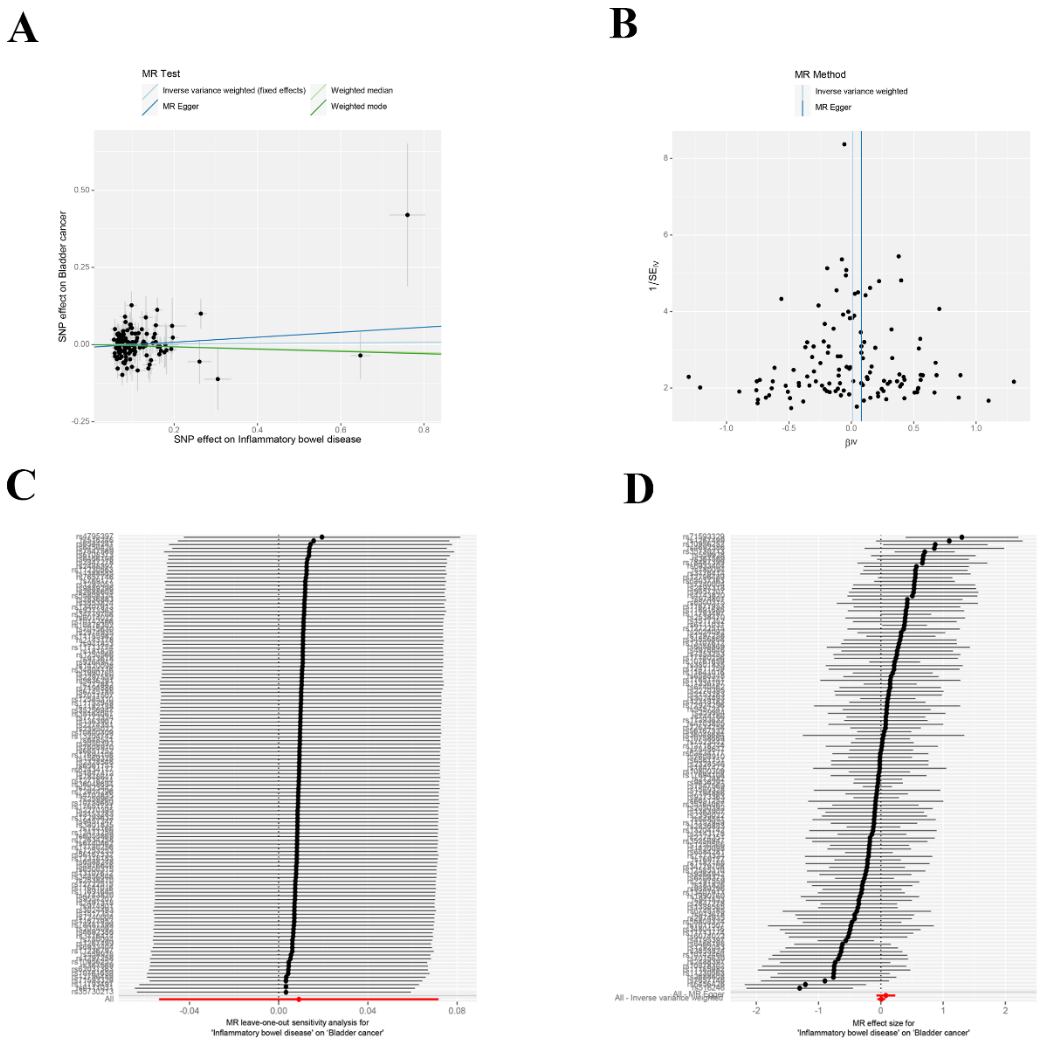

Figure S4. Scatter plot (A), funnel plot (B), leave-one-out sensitivity analysis (C), forest plot (D) of the causal effect of IBD on BC risk based on FinnGen.

Supplementary Figure S5.

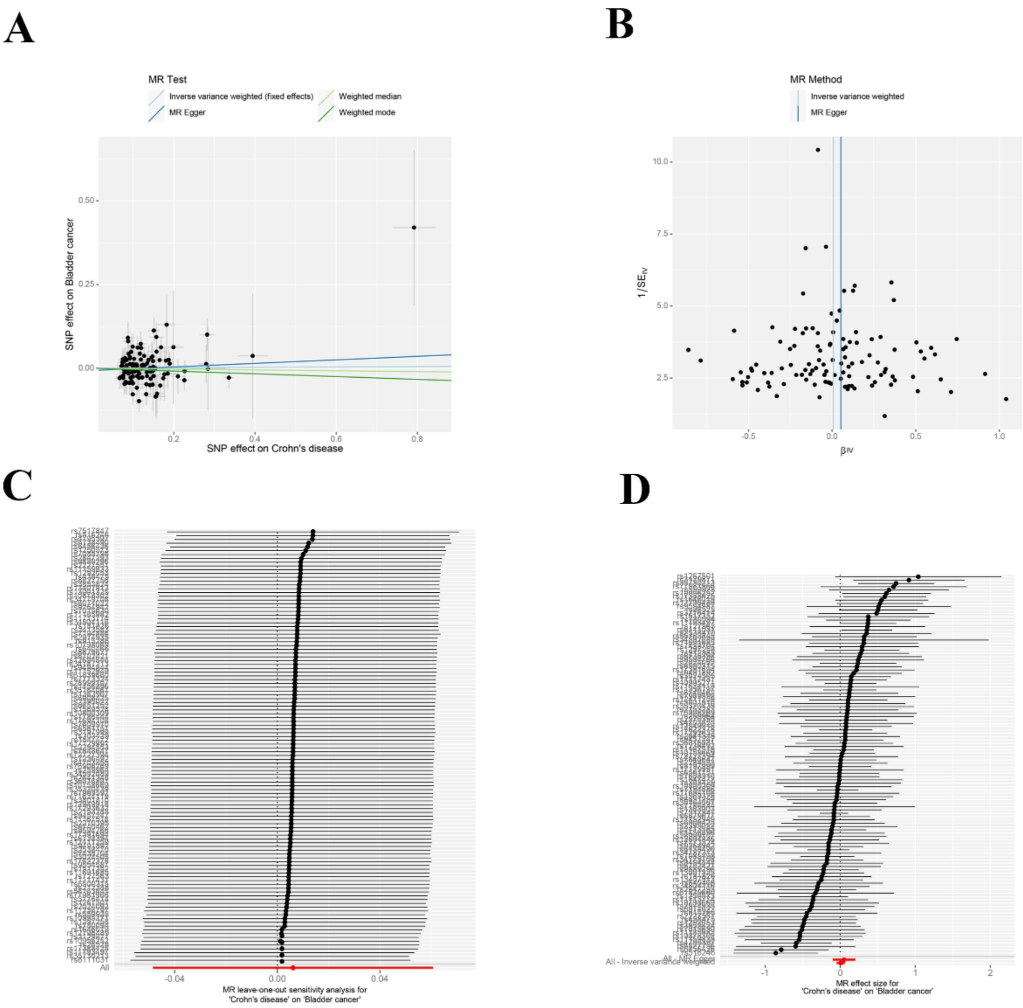

Figure S5. Scatter plot (A), funnel plot (B), leave-one-out sensitivity analysis (C), forest plot (D) of the causal effect of CD on BC risk based on FinnGen.

Supplementary Figure S6.

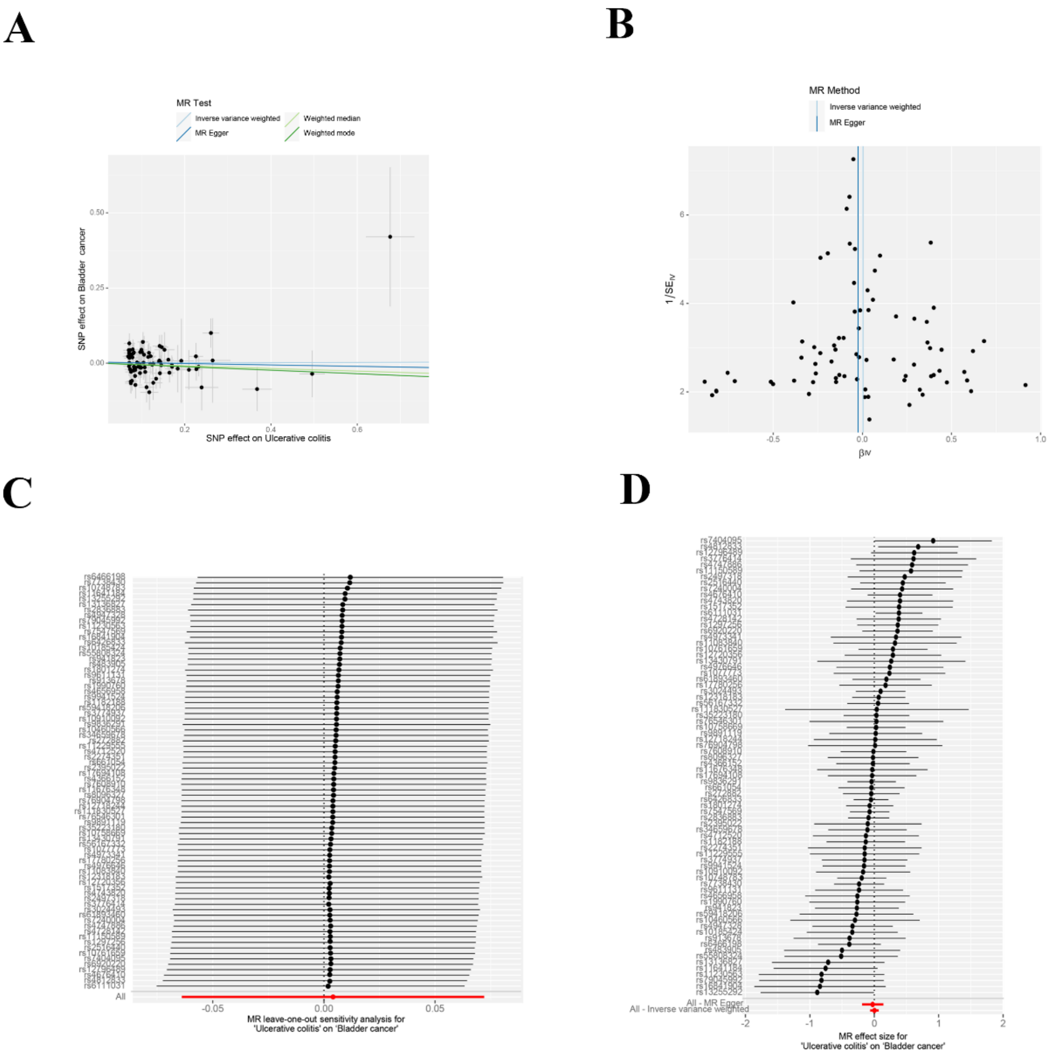

Figure S6. Scatter plot (A), funnel plot (B), leave-one-out sensitivity analysis (C), forest plot (D) of the causal effect of UC on BC risk based on FinnGen.
